# Plantago Major for Bite of Rattlesnae

**Published:** 1864-05

**Authors:** Wm. Foster

**Affiliations:** M. Student; Buffalo


### PLANTAGO MAJOR FOR BITE OF RATTLESNAE.

To the Editor of the Buffalo Medical and Surgical Journal:

Dear Sir:—I submit to you the following case of a rattlesnake bite in a dog, treated by the external and internal use of the expressed juice of *plantago major.* If you think it would be of any interest to the readers of your worthy Journal, you may give it to them in this or any other form your more mature judgment approves.

In the summer of 1861, whilst attending to some business in my field about one-fourth of a mile from the town of Welland, Canada West, a Mr. Hooker of the same town came over to see me, and he had his dog with him, a beautiful hound, and very highly prized by his master. We were but a few minutes in conversation when our attention was called to what we then thought was an engagement between the hound and some animal of the same kind; but when we reached the scene of action, we found that it was a large rattlesnake. He dispatched the snake, but not until he had received several bites on the snout and head.

I must confess that although I would have been very sorry had Mr. Hooker lost his dog, I was not extremely so to have the chance of testing the power of remedies, where it was impracticable either to destroy or remove the parts. He concluded to do nothing to him until we saw the virus begin to take effect, (a wrong principle, except for the sake of experiment, and that on a canine subject,) but we had not long to wait, for he soon began to manifest symptoms of dullness and listlessness, and his head began to swell. We then took him to Mr. Stones, about eighty rods from where it happened, and Mr. S. being a great man for herbs, got some plantain, bruised it, expressed the juice, and gave him as much as he could of it internally, also applied it externally. By this time the dog's head was so much swollen as to make it impossible to see his eyes. And the constitutional effect was such as to make his walk as zig-zag as that of a certain species of bipeds when bitten by the serpent of the still. In less than an hour the swelling began to subside, but he was very weak. In about three hours he followed his owner home, a distance of half a mile. Next day he was running around town as well as ever.

Yours, most respectfully, WM. FOSTER, M. Student.

*Buffalo, April* 28, 1864.

